# Communication Outcomes of Children with Hearing Loss: A Comparison of Two Early Intervention Approaches

**DOI:** 10.3390/audiolres15020027

**Published:** 2025-03-08

**Authors:** Aisha Casoojee, Katijah Khoza-Shangase, Amisha Kanji

**Affiliations:** Department of Audiology, Faculty of Humanities, School of Human and Community Development, Braamfontein East Campus, University of the Witwatersrand, Johannesburg 2000, South Africa; katijah.khoza-shangase@wits.ac.za (K.K.-S.); amisha.kanji@wits.ac.za (A.K.)

**Keywords:** auditory–verbal therapy, early hearing detection and intervention, hearing loss, listening and spoken language, South Africa, communication outcomes, speech-language therapy, inclusive education

## Abstract

Background: Early intervention approaches play a critical role in shaping the communication outcomes of children with hearing loss, influencing their language development and overall learning trajectory. Objectives: The main objective of this study was to compare the communication outcomes of children with hearing loss who received Listening and Spoken Language-South Africa (LSL-SA) with those who received Traditional Speech-Language Therapy (TSLT). Methods: A retrospective record review was conducted to gather data on communication outcomes from participants’ speech-language therapy records. Communication outcomes were measured using standardized assessments evaluating speech intelligibility, expressive vocabulary, receptive language, expressive language, audition, and cognitive–linguistic skills. The data were analyzed using quantitative statistics. Key statistical methods included measures to determine associations, identify statistical significance, determine outcomes, and compare differences between the two groups. Results: The study found that children in the LSL-SA group had statistically significant better communication outcomes, with 63% achieving age-appropriate speech intelligibility compared to 45% in the TSLT group (*p* = 0.046). Similar trends were observed for expressive vocabulary (LSL-SA: 58% vs. TSLT: 39%, *p* = 0.048) and receptive language (LSL-SA: 60% vs. TSLT: 39%, *p* = 0.043). Additionally, 66% of children in the LSL-SA group were recommended for mainstream schooling, compared to 39% in the TSLT group (*p* = 0.0023). These findings highlight the importance of early amplification and structured intervention in improving communication outcomes. The results also emphasize the importance of Early Hearing Detection and Intervention (EHDI) in decreasing the odds of delay in communication outcomes, irrespective of the type of communication approach, although a higher proportion of children in the LSL-SA approach group achieved age-appropriate communication outcomes than those in the TSLT group. Conclusions: This study highlights that communication intervention approaches aligned with the LSL-SA practice promote better communication development and enhance spoken language outcomes in children with hearing loss, facilitating successful transitions to mainstream schooling. Contribution: This study provides contextually relevant evidence for implementing an LSL-SA intervention approach for children with hearing loss. The implications of these findings for clinical practice and future research are discussed in detail.

## 1. Introduction

Congenital or early-onset hearing loss poses significant challenges in children, impacting the development of communication skills, cognitive abilities, psychosocial skills, and academic outcomes [1,2,3]. Consequently, the Joint Committee on Infant Hearing in the United States proposed an Early Hearing Detection and Intervention (EHDI) program to maximize the linguistic competence of infants and young children with hearing loss [4]. Similar policies and guidelines exist globally, including the European Consensus Statement on Neonatal Hearing Screening and the World Health Organization’s recommendations for early hearing intervention, highlighting the widespread recognition of EHDI as a public health priority.

Components of an EHDI program include (1) newborn hearing screening that aims to identify infants with hearing loss, ideally by one month of age, (2) audiologic diagnosis to confirm the presence and degree of hearing loss for infants who fail the initial hearing screening by three months of age, and (3) referral to early intervention (EI) services by six months of age [4]. EI services include audiological management, educational support, family counselling, and communication intervention.

Communication intervention aims to support children with hearing loss and their families during the critical stages of language development, typically from birth to three years of age. During this period, the brain undergoes rapid growth and plasticity, making it particularly receptive to language input [5]. Numerous studies have shown EI to significantly improve communication outcomes in children with hearing loss across domains that include speech intelligibility, expressive vocabulary, receptive language, expressive language, audition, and cognitive linguistics (cognitive linguistics informs AVT how children with hearing loss process auditory information to support language, attention, memory and conceptual thinking) outcomes [6,7,8,9,10,11,12]. The interventions explored in this study, while effective in promoting spoken language outcomes, must be critically examined within the context of systemic inequities that limit access to alternative communication modalities, such as South African Sign Language (SASL). This focus on spoken language risks marginalizing Deaf cultural identity and the right to linguistic diversity, as emphasized by the CRPD. Positive communication outcomes are limited to studies using the 1-3-6 benchmark by which children with hearing loss were diagnosed and received amplification and EI (i.e., 1-3-6 EHDI time-frame benchmark) [13,14,15,16,17,18,19,20,21]. Whilst this benchmark is recommended, it has not been fully achieved in South Africa due to delays in identification and early intervention [3,22]. Within these proposed timeframes, there are important variables to consider as possible factors contributing to communication outcomes. These variables include the hearing amplification device fitted, the degree of the aided audiogram, the communication intervention approach adopted, and linguistic diversity influences on outcomes [23]. While this study focuses on spoken language approaches, we acknowledge the importance of Deaf cultural identity and the validity of sign language as a primary mode of communication. Future research and interventions should aim to balance linguistic, cultural, and individual preferences to foster inclusivity.

South Africa, a low-middle-income country (LMIC), has lagged in implementing EHDI as a standard [3]. Currently, more than 90% of infants with hearing loss are not identified early or provided with intervention, largely due to (1) budgetary constraints affecting health policy, (2) limited access to services, (3) poor-quality Early Childhood Development (ECD) structures, and 4) a lack of comprehensive empirical evidence [3,24,25]. These systemic barriers prevent families from making fully informed choices about communication modalities, which include spoken language, sign language, or bilingual approaches.

Hearing loss is recognized as a disability under the social and rights-based models of disability [26,27]. This perspective acknowledges that barriers to effective communication and language acquisition can lead to social exclusion, educational challenges, and limited future opportunities for individuals with hearing loss. In this study, EI approaches (TSLT and Listening and Spoken Language-South Africa [LSL-SA—based on the LSLS Certified Auditory–Verbal Therapist (LSLS Cert. AVT^®^) qualification by the AG Bell Academy [24] are analyzed for their potential to support inclusive education and enhance life outcomes for children with hearing loss, aligning with the goals of disability frameworks that emphasize reducing barriers, promoting equity, and enabling full societal participation. This positioning underlines the importance of evidence-based interventions that improve communication skills, thereby fostering access to mainstream schooling and social integration, essential components of inclusive development within disability frameworks.

Studies on communication intervention approaches for children with hearing loss have primarily taken descriptive approaches [28]. Roberts [28] has found that, despite details on the type of intervention approach being included in these studies, these studies either observe convenience samples of children undergoing specific interventions [29,30] or track children participating in EI programs longitudinally [12,18]. There is a scarcity of studies comparing the outcomes under different communication intervention approaches. Results of a study conducted by Percy-Smith et al. [31] indicated that children with hearing loss who enrolled in an LSL approach scored age-appropriately and outperformed children with hearing loss enrolled in a TSLT approach.

In this study, two intervention approaches were compared: Traditional Speech-Language Therapy (TSLT) and Listening and Spoken Language—South Africa (LSL-SA). TSLT is a multimodal approach incorporating auditory training, speech production exercises, and visual supports such as lip-reading and gestures, and is grounded in a general speech-language pathology framework, focusing on enhancing auditory perception, speech production, and language skills, typically involving a mix of auditory and visual supports tailored to the child’s needs. The TSLT approach typically incorporates multimodal communication strategies, including auditory training, speech articulation exercises, and visual cues such as lip-reading and gestures. Therapy sessions are structured to address individualized goals, often with periodic assessments to adapt the intervention plan based on the child’s progress. TSLT sessions also include strategies for language expansion through interactive play and storytelling to support vocabulary and sentence structure development. In contrast, LSL-SA follows an AVT model, which is highly parent-centered and focuses on developing auditory skills in everyday contexts. The AVT model emphasizes auditory input over visual cues to develop spoken language, based on the principle that consistent auditory access can maximize language outcomes for children with hearing loss. Intervention involves teaching parents to create rich auditory learning environments at home, promoting the consistent use of hearing devices such as cochlear implants or hearing aids. Sessions are intensive and structured, emphasizing active listening skills, phoneme discrimination, and sentence-level auditory comprehension. Visual cues are minimized to encourage auditory reliance, with therapists’ modeling techniques to integrate speech and language goals into routine family interactions. Both approaches aim to develop age-appropriate spoken language skills, though they differ in their theoretical emphasis on auditory vs. multimodal (auditory and visual) support.

These interventions are individualized, considering factors such as the child’s degree (mild to profound) and type (sensorineural, conductive, or mixed) of congenital hearing loss, age at diagnosis, and age at intervention. LSL-SA may be more intensive and parent-centered, involving regular family participation to integrate language learning into everyday environments, whereas TSLT may employ a broader range of communication supports. In evaluating outcomes, both approaches assess speech intelligibility, expressive and receptive language, vocabulary, and cognitive–linguistic skills.

However, while this study compares TSLT and LSL-SA, both focus on spoken language development but diverge from Deaf cultural identity and the use of SASL. This comparison highlights a broader debate in communication sciences and disorders: the prioritization of spoken language versus sign language and the implications of these choices for Deaf identity and inclusion within Deaf communities [32]. While the current study primarily focuses on spoken language outcomes, it highlights the need for future research to explore the broader implications of Deaf identity and cultural alignment of intervention approaches, especially in LMIC contexts where family-centered and linguistically congruent options are limited [23,32].

This study also aligns with current debates around Deaf identity and the choice of Sign Language as a primary communication mode, particularly in contexts where LSL approaches may not align with the cultural and linguistic preferences of Deaf communities. SASL offers an equally valid communication option that fosters a strong Deaf cultural identity. However, due to the limited availability of SASL resources, particularly in LMICs, spoken language approaches may be the only accessible intervention option for many families [32]. Future research should consider integrating Deaf cultural perspectives and increasing access to bilingual or bimodal communication options in early intervention to foster more holistic and culturally responsive outcomes.

The National Development Plan (NDP) 2030 for Persons with Disabilities (2015) seeks to address the needs of children with disabilities, including children with hearing loss, who are at risk of being excluded from early childhood investments in South Africa [33]. The NDP aims to roll out a process involving inclusive education and the implementation of continuity of care between EI programs and educational settings, dependent on proper and effective monitoring and evaluation systems that track progress against goals and objectives [33,34]. This roll-out was envisaged to improve the access to and quality of ECD services for infants and young children. However, educational intervention for children with hearing loss is already at risk, given the paucity of resources accessible to these children [3,35]. Of the 43 Schools for the Deaf, only 12 offer education up to the National Senior Certificate level, which is a pre-requisite for entering any institution of higher learning [36,37,38]. This limited access undermines efforts to build an inclusive society [24].

If left unaddressed, hearing loss can have severe consequences, including delayed communication development, leading to poor academic outcomes and, consequently, fewer employment opportunities, personal–social maladjustments, and emotional difficulties [39]. To counteract these realities, it is imperative that efforts aimed at EHDI are maximized and that strategies facilitating positive outcomes from these processes are investigated. The current study was prompted by findings from a previously conducted exploratory study investigating the learning outcomes of foundation phase learners with hearing loss. Whilst these findings also included speech and language outcomes as part of the learning outcomes and suggested the LSL-SA approach as having maximized linguistic competence and literacy development of children with congenital or early-onset hearing loss, the sample of records was small and limited to EI schools in three provinces in South Africa [24]. The current study proffers additional insight, providing a trajectory into the communication outcomes using a larger, more representative sample of records from EI schools across four provinces in South Africa. While this study focuses on South Africa, similar challenges exist in many LMICs, as well as underserved regions in high-income countries (HICs). Limited access to newborn hearing screening, delays in diagnosis and intervention, and disparities in service provision have been reported in countries such as Brazil, India, and rural parts of the United States. These global challenges highlight the need for contextually relevant early intervention approaches that can be adapted across diverse healthcare and educational settings.

Therefore, the main aim of this study was to investigate the communication outcomes (i.e., speech intelligibility, expressive vocabulary, receptive language, expressive language, audition, cognitive linguistics) of children with hearing loss who received LSL-SA versus TSLT in South Africa. The specific objectives included (1) describing the communication functioning prior to the commencement of EI and upon discharge from therapy in both the LSL-SA group and TSLT group; (2) describing the communication functioning prior to the commencement of EI and upon discharge from therapy in the LSL-SA and TSTL groups; (3) comparing the communication outcomes between the two groups; (4) determining if any associations exist between selected study variables (i.e., age at identification of hearing loss, age at diagnosis of hearing loss, age at amplification, duration of amplification, hearing amplification device, age at start of EI, duration of EI, degree of the aided audiogram, home language vs. language used in therapy) within the communication intervention approaches and communication outcomes; and (5) identifying the type of school recommended after EI, based on communication outcomes.

## 2. Materials and Methods

### 2.1. Study Design

A retrospective record review was conducted to compare communication outcomes in children with hearing loss who received either Listening and Spoken Language-South Africa (LSL-SA) or Traditional Speech-Language Therapy (TSLT). The study used quantitative data analysis to assess associations between intervention type and communication outcomes. As this was a non-randomized study, children were grouped based on the intervention they had received within their educational setting, and no manipulation of intervention assignment was performed. While the children with hearing loss in the study received only one intervention (either LSL-SA or TSTL), the type of therapy was determined by the school and not by the researcher. Thus, the assignment to LSL-SA or TSTL was not controlled but rather a reflection of the services available within each school setting at the time of the child’s intervention.

### 2.2. Study Objectives and Corresponding Variables

The study aimed to achieve the following objectives, with each linked to the data collected:

1. Describe communication functioning prior to and upon discharge from early intervention (EI) for both the LSL-SA and TSLT groups.
  ○ *Variables:*
    ▪ Communication Functioning: speech intelligibility, expressive vocabulary, receptive language, expressive language, audition, cognitive–linguistic skills (all ordinal variables: delayed, age-appropriate).

2. Compare communication outcomes between the LSL-SA and TSLT groups.
  ○ *Variables:*
    ▪ Type of Communication Intervention: TSLT OR LSL-SA (nominal).
    ▪ Outcome Measures: proportion of children with age-appropriate communication skills at discharge (ordinal).

3. Determine associations between selected study variables and communication outcomes within each intervention approach.
  ○ *Variables:*
    ▪ Age at Identification of Hearing Loss (ratio, years).
    ▪ Age at Diagnosis of Hearing Loss (ratio, years).
    ▪ Age at Amplification (ratio, years).
    ▪ Type of Amplification Device (hearing aid or cochlear implant, nominal).
    ▪ Age at Start of EI (ratio, years).
    ▪ Duration of EI (ratio, years).
    ▪ Degree of Aided Audiogram (ordinal: normal, mild, moderate, severe).
    ▪ Home Language vs. Language Used in Therapy (nominal: matched or not matched).

4. Identify the type of school recommended after EI based on communication outcomes.
  ○ *Variables:*
    ▪ Type of School Recommended (nominal: mainstream or specialized).

### 2.3. Study Sample and Demographics

This study reviewed records from 126 children with profound congenital or early onset hearing loss attending EI schools across four South African provinces: Western Cape (Cape Town), Gauteng (Johannesburg and Pretoria), KwaZulu Natal (Morningside), and Eastern Cape (Gqeberha). These schools serve diverse linguistic and socioeconomic populations, with participants speaking five of South Africa’s official languages. Many families come from low- to middle-income communities with limited access to private healthcare, relying on public sector EI services. Most participants received communication intervention in English, with some receiving intervention in Afrikaans. The study timeframe spanned records collected over a five-year period, allowing a comprehensive assessment of communication outcomes for children allocated to either a TSLT or LSL-SA intervention approach. The EI schools offer both therapy options; however, each child is allocated to only one type of intervention.

### 2.4. Inclusion and Exclusion Criteria

Records of participants who met the following criteria were included: (1) had a diagnosis of congenital or early onset, bilateral, sensorineural, profound hearing loss; (2) were enrolled in an EI program receiving either TSLT or the LSL-SA approach; (3) had cochlear implants or were fitted with hearing aids (as approved by a South African Cochlear Implant Group-endorsed cochlear team); and (4) had finished Grade 3 in the foundation phase of primary schooling. Participants were assessed at discharge from EI, typically between the ages of 6 and 10 years, ensuring comparability between groups. Developmental factors, including age at identification, age at amplification, and duration of intervention, were accounted for in the analysis to minimize underlying variability. The study excluded children with unilateral profound hearing loss or bilateral profound hearing loss who also had other documented co-morbidities, such as cognitive impairment, to ensure a homogenous sample focused on the effects of intervention alone.

### 2.5. Procedures

Ethical approval from the University of the Witwatersrand Ethics Committee (HREC) (protocol number: H20/06/03). Thereafter, permission was sought from all relevant authorities, granting the researcher access to the educational facilities and disseminating information to the parents/primary caregivers of children with hearing loss. Written informed consent was obtained from the parents/primary caregivers of children with hearing loss after indicating their willingness to allow the researcher to access their child’s records. Once permission and consent were obtained, records were accessed and reviewed. As this study was retrospective in nature, it was reliant on pre-existing records, which may not uniformly capture all variables of interest. Consequently, the findings are descriptive and associative rather than causative, and the conclusions drawn must be interpreted with caution.

### 2.6. Data Collection Tool

The data collection tool used in the current study was specifically designed for this purpose. It was pre-tested on a therapy file from each EI school before use. None of the records used in the pre-test phase formed part of the main study. The data collection form consisted of two sections. Section A gathered information on child demographics, and Section B gathered information on Intervention Outcomes. The record review aimed to gather information regarding (1) the child’s hearing loss, (2) the age at diagnosis of hearing loss and age at initiation of EI services, (3) the type of amplification device fitted and duration of use, (4) the type of communication intervention approach, and (5) speech-language communication functioning at the onset of therapy and communication outcomes at the end of grade three at the EI school and (6) the type of school recommended upon completion of the foundation phase of primary school. This study compares specific approaches but does not negate the relevance of alternative communication methods, including sign language, which may align more closely with the values of Deaf communities.

Communication functioning was measured consistently across all EI schools using standardized assessment tools to evaluate domains including speech intelligibility, expressive vocabulary, receptive language, expressive language, audition, and cognitive–linguistic skills. These assessments were administered by qualified speech-language therapists at the beginning of therapy and upon discharge, ensuring uniformity in evaluating communication progress. Data were collected retrospectively over a five-year period, providing a longitudinal view of each child’s progress. Records were detailed, capturing information on the initial communication functioning at therapy onset, intermediate progress notes, and final outcomes, allowing for a comprehensive analysis of communication development in each intervention group.

### 2.7. Standardized Assessment Tools

The communication outcomes measured in this study—including speech intelligibility, expressive and receptive language, vocabulary, audition, and cognitive–linguistic skills—were assessed using validated tools commonly used in South Africa. These included the Preschool Language Scale—Fifth Edition (PLS-5), Clinical Evaluation of Language Fundamentals, Integrated Scales of Language Development, Functional Listening Index-Paediatric, and the Goldman Fristoe Test of Articulation, which have been adapted and used in multilingual South African contexts. Assessments were conducted by qualified speech-language therapists, ensuring standardized administration across all early intervention (EI) sites.

### 2.8. Data Analysis

Each research question was analyzed using the following models:

- Objectives 1 and 2: Comparing Communication Functioning and Outcomes:

Descriptive statistics (means, standard deviations, percentages) were used to summarize communication outcomes in both groups. Comparisons were conducted using independent samples *t*-tests for continuous variables and chi-square tests for categorical variables. For categorical variables with small cell sizes, Fisher’s exact test was used instead of the chi-square test.

- Objective 3: Associations Between Study Variables and Communication Outcomes:

Logistic regression models were used to assess associations between intervention type (LSL-SA vs. TSLT) and outcome measures (age-appropriate vs. delayed) while controlling for confounding variables (age at amplification, device type, duration of EI). For model transparency, logistic regression outputs after multiple comparisons, significance levels were set at *p* < 0.021.

- Objective 4: School Placement After EI:

Associations between communication outcomes and the type of school recommended were assessed using Fisher’s exact test.

All data were analyzed using SAS version 9.4 for Windows [40]. Blinding in data analysis occurred. To minimize bias, the researchers conducting the data analysis were blinded to the intervention group assignments during statistical processing. Data were de-identified and coded before analysis to ensure that the analysts were unaware of whether participants had received LSL-SA or TSLT. This approach enhanced the objectivity and validity of the findings by preventing any potential influence of preconceived expectations on the interpretation of results.

### 2.9. Ethical Considerations

This study was conducted in accordance with ethical guidelines for research involving human participants. Ethical approval was obtained from the University of the Witwatersrand Human Research Ethics Committee (HREC) (Protocol Number: H20/06/03) before data collection commenced. Permission was also granted by relevant educational authorities and school administrators to access speech-language therapy and audiology records. As this was a retrospective record review, informed consent was obtained from the parents or primary caregivers of all children whose records were included in the study. To ensure confidentiality and data protection, all records were anonymized, with unique identification codes assigned to participants instead of personal identifiers. Data were stored in a password-protected database accessible only to authorized research personnel. The study adhered to the principles outlined in the Declaration of Helsinki, ensuring respect for participant privacy, non-maleficence, and responsible data handling. Findings were reported objectively and without bias, with appropriate caution taken to acknowledge study limitations related to its retrospective and non-randomized design.

### 2.10. Validity and Reliability

To ensure validity and reliability, this study employed standardized data collection methods and used validated assessment tools to measure communication outcomes across all EI sites. Content validity was maintained by aligning outcome measures—such as speech intelligibility, expressive and receptive language, and auditory skills—with established early intervention frameworks. Reliability was enhanced through consistent administration of assessments by qualified speech-language therapists and audiologists, minimizing inter-rater variability. Additionally, statistical controls were applied to account for potential confounders, such as age at amplification and duration of intervention, strengthening the internal validity of findings. External validity was considered by including a diverse sample from multiple provinces, making the findings more generalizable to similar contexts within South Africa and other LMICs. Data accuracy and consistency were ensured through rigorous double-checking procedures during data entry and statistical analysis. These methodological safeguards support the trustworthiness and reproducibility of the study’s results.

### 2.11. Data Management

Data management involved collecting, storing, and securing participant records to ensure the integrity and confidentiality of sensitive information. Data were sourced from retrospective records at EI schools, and each record was anonymized by assigning unique identification codes to participants to protect their identities. The data were transferred to a secure, password-protected database accessible only to authorized research team members. Variables collected included demographic information, communication outcomes, intervention details, and audiological data, which were carefully entered and checked for accuracy and completeness. Regular data-cleaning addressed inconsistencies or missing information. All data handling complied with ethical guidelines, ensuring that participants’ privacy and data security were maintained throughout the study. After analysis, data were securely archived for future reference while retaining confidentiality standards.

## 3. Results

### 3.1. Demographic Profile of Participants

As depicted in Table 1, of the 126 participants, 64 were enrolled in TSLT, and 62 were enrolled in the LSL-SA group. Both groups had more females than males, with ages ranging between 6.0 and 10.1 years old. Participants spoke five of South Africa’s 12 official languages and represented all four recognized race groups [41]. In the TSLT group, 41 participants wore cochlear implants, compared to 48 participants in the LSL-SA group, with 22 participants in the TSLT group and 29 in the LSL-SA group using bilateral cochlear implants.

Genetic causes of hearing loss accounted for 14% of participants, prenatal infections for 17%, and postnatal infections for 18%. The cause of hearing loss remained unknown in 51% of participants due to missing data.

### 3.2. Communication Functioning Prior to the Commencement of EI and upon Discharge from Therapy in the TSLT and LSL-SA Groups

#### 3.2.1. The Description of Communication Functioning Prior to the Commencement of EI to the Communication Outcomes upon Discharge from Therapy in the TSLT Group (n = 64) Is Depicted in Table 2

At the onset of therapy, 98% (n = 63) of children in the TSLT group exhibited delayed communication skills across all domains, including speech intelligibility, expressive vocabulary, receptive language, expressive language, audition, and cognitive linguistics. Of these, 36% (n = 23) were fitted with hearing aids, and 64% (n = 41) had cochlear implants. Upon discharge from therapy, 39% to 48% of participants showed improvements, achieving age-appropriate communication skills across all the domains.

#### 3.2.2. The Description of Communication Functioning Prior to the Commencement of EI to the Communication Outcomes upon Discharge from Therapy in the LSL-SA Group (n = 62) Is Depicted in Table 2

Results indicate delayed communication skills in speech intelligibility, expressive vocabulary, receptive language, expressive language, audition, and cognitive–linguistic skills ranging from 90% to 98%. Of the participants in the LSL-SA group, 23% (n = 14) were fitted with hearing aids, and 77% (n = 48) had cochlear implants. Upon discharge from therapy, significant improvements were observed in achieving age-appropriate communication skills ranging from 56% to 73% across all the communication domains.

### 3.3. Comparing the Communication Outcomes Between the Two Groups

Comparing the communication outcomes between the two groups (i.e., TSLT and LSL-SA), as depicted in Table 3, the categories for “age-appropriate—delayed” and “age-appropriate—age-appropriate” were very small and were grouped into categories “delayed—delayed” and “delayed—age-appropriate”, respectively.

A comparison of outcomes between TSLT and LSL-SA was conducted and adjusted for significant differences in characteristics between the two groups, namely age at identification and age at amplification. The analysis also accounted for usual development, which reflects the natural progression of children over time, by considering the duration of early intervention (EI), as children typically develop and change during this period. Although the duration of EI did not differ significantly between the groups, it was included in the analysis as a post-hoc measure to assess its impact on communication outcomes. The results from this post-hoc analysis indicated that longer durations of EI were associated with reduced delays in communication development.

Findings further indicate statistically significantly decreased odds of remaining or becoming delayed in speech intelligibility and expressive vocabulary for the LSL-SA group (compared to the TSLT group). There were statistically significant decreased odds of remaining or becoming delayed in receptive language, audition, and cognitive–linguistics, both unadjusted and adjusted for the earliest age of amplification for the LSL-SA group (compared to the TSLT group). Findings further indicate that children with hearing loss who underwent LSL-SA were characterized by a higher proportion of participants who attained age-appropriate speech intelligibility, expressive vocabulary, receptive language, audition, and cognitive–linguistics upon discharge from therapy compared to those in TSLT.

### 3.4. Determining If Any Associations Exist Between Selected Study Variables and Communication Outcomes Within Each Communication Intervention Approach

Findings on the selected study variables of participants (i.e., age at identification of hearing loss, age at diagnosis of hearing loss, age at amplification, duration of amplification, hearing amplification device, age at start of EI, duration of EI, degree of aided audiogram, home language vs. language used in therapy) within the intervention approaches, are depicted in Table 4.

When examining the age of identification, diagnosis, and fitting of amplification devices for children with hearing loss between the LSL-SA (n = 62) and TSLT (n = 64) groups, findings revealed that participants in the LSL-SA group were identified and diagnosed with hearing loss at younger ages and fitted with hearing amplification devices at a younger age compared to those in the TSLT group. These findings show that participants in the LSL-SA group have earlier access to EHDI (identification = 1.0 years old, diagnosis = 1.5 years old, amplification = 2.3 years old) services, leading to earlier intervention for children with hearing loss than those in the TSLT group.

There is no statistically significant time difference between the LSL-SA and the TSLT groups when comparing the time taken from diagnosis of hearing loss to the start of communication intervention. The study also examined the average duration of communication intervention sessions attended by participants, averaging 4.6 years; notably, participants in the LSL-SA group (n = 62) attended therapy for an average of 4.2 years, significantly less than those in the TSLT group (n = 64), who attended for an average of 5.5 years.

In both the TSLT and LSL-SA groups, the majority of participants received communication intervention in English. A subset of participants, 33% (n = 21) in TSLT and 26% (n = 16) in LSL-SA received intervention in Afrikaans. Of these, 78% (n = 50) in TSLT and 81% in LSL-SA received intervention in their home language. The minority, 22% (n = 14) in TSLT and 19% (n = 12) in LSL-SA, received intervention in a language other than their home language.

Table 5 depicts the aided audiogram results between the TSLT group (n = 44) and the LSL-SA group (n = 22). The audiological assessment measures were conducted by registered audiologists (i.e., Health Professions Council of South Africa [HPCSA]) using calibrated equipment (i.e., SANS 10182:2006) in a calibrated soundproof booth that complies with standards and regulations (i.e., S EN ISO 8253-1: 2010) [42,43]. Participants who failed to achieve normal aided thresholds across all frequencies had aided audiograms within the mild hearing loss category in both groups.

Table 6 depicts the associations that exist between the selected study variables (i.e., age at identification of hearing loss, age at diagnosis of hearing loss, age at amplification, duration of amplification, hearing amplification device, age at start of EI, duration of EI, degree of aided audiogram, home language vs. language used in therapy) and the communication outcomes (controlling for the communication intervention approach and hearing amplification device). No statistically significant association was found between the communication intervention approach and study variables, except that those who received LSL-SA had a lower median age of identification and a lower median earliest age of amplification. These factors were adjusted when comparing outcomes between TSLT and LSL-SA, as the other variables were most likely not prognostic for outcomes or were themselves outcomes. Since age of identification and earliest age of amplification were strongly correlated (r = 0.816; *p* < 0.0001), only the earliest age of amplification was used.

**Figure 1:**
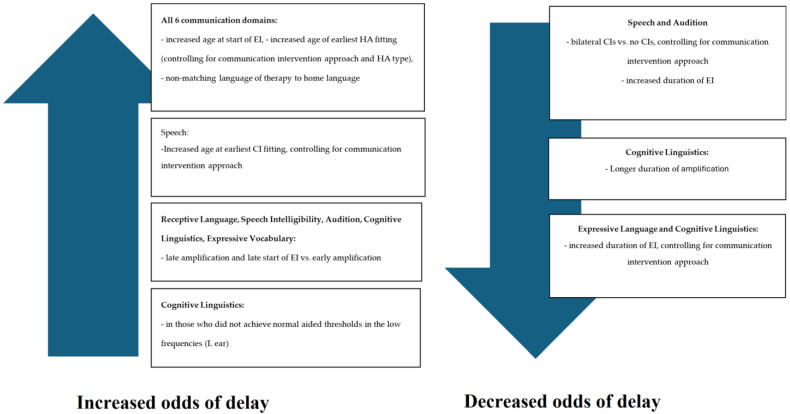
Statistically significant associations between variables result in increased or decreased odds of delay.

Figure 1 below depicts the association between the selected variables and communication outcome (controlling for TSLT or LSL-SA and hearing amplification device). It presents the statistically significant associations that result in increased or decreased odds of delay across the six communication domains, after correction for multiple comparisons.

### 3.5. Identifying the Type of School Recommended After EI Based on Communication Outcomes

**Figure 2:**
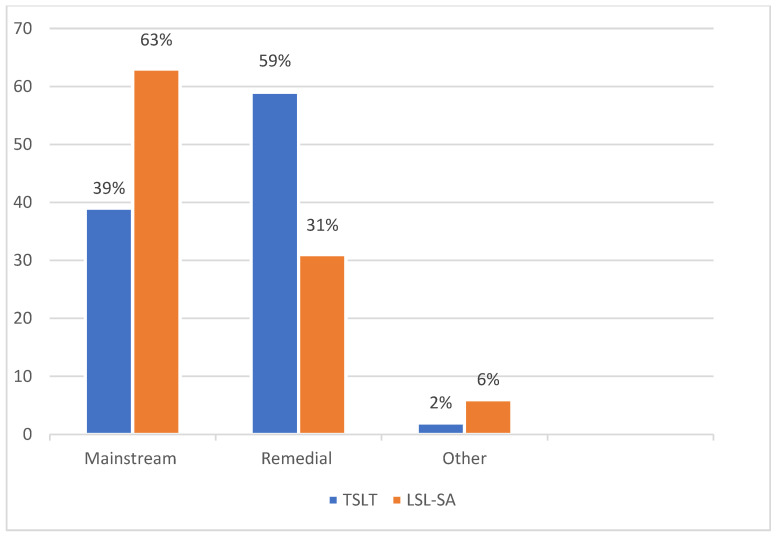
Type of school recommended upon discharge from communication intervention.

When examining the type of school recommended after EI based on communication outcomes, as depicted in Figure 2, the participants who received LSL-SA (n = 25) had a higher proportion of being recommended for mainstream schooling in comparison to the participants who received TSLT (n = 39) (*p*-value for between-group comparison = 0.0023), who were recommended for specialized schooling.

## 4. Discussion

The sample comprised the speech-language therapy records of 126 children with hearing loss enrolled at one of four participating EI schools in South Africa. All the children were discharged from communication intervention therapy at the end of their foundation phase of schooling. The study affirms the validity of the speech-language outcomes measures, as consistent communication assessment tools were used across the EI schools to evaluate these outcomes. Given the retrospective nature of the study, which relied on existing records, slight variations in record-keeping and assessment practices were considered. Although standardized assessment tools were used, the potential impact of these variations on the consistency and validity of the outcomes was taken into account in the analysis.

Research confirms multiple advantages to hearing with two cochlear implants [44,45]. Despite the provision of free hearing aids to children under six and a national CI platform, rehabilitative interventions compete with life-threatening conditions within an over-stressed and under-resourced health budget and are poorly prioritized [3,46]. In 2019, only 23 children received government-funded CIs despite 17 babies born daily with permanent sensorineural hearing loss in South Africa [46,47]. If hearing amplification devices are available and used optimally (i.e., appropriate pre-fitting counselling, device management skills, and post-fitting support), it would ensure better health and developmental outcomes for children with hearing loss in South Africa.

Findings regarding the aetiology of hearing loss identified in this study correlate with research conducted by Shearer et al. [48], Korver et al. [49] and Mulwafu et al. [50]. In accordance with the exploratory study [51], the risk profile of infants in an LMIC has been highlighted, raising implications in the planning of EHDI in the South African context.

When describing the communication functioning prior to the commencement of EI to the communication functioning upon discharge from therapy in the TSLT group, findings indicate that 98% of participants had delayed communication skills, consistent with the literature which suggests auditory deprivation during the first three years of life is linked to less favorable outcomes in speech intelligibility, language, developmental, educational, and cognitive outcomes in children [52,53,54,55]. Upon discharge from therapy, only 48% of participants achieved age-appropriate communication skills. This is consistent with the findings of Lederberg et al. [56] that the majority of children with profound hearing loss in communication interventions, such as sign language and sign-supported approaches, do not achieve age-appropriate grammar, language-related areas of development (i.e., theory of mind) and literacy development, compared to their normal hearing peers, despite early identification. The current study’s findings also concur with the study by Harris et al. [57], comparing the language and literacy of two cohorts of children with profound hearing loss enrolled in TSLT, recruited ten years apart. Harris et al. [57] reported that, while the second cohort had a higher vocabulary score, this was not matched by commensurate phonological awareness or reading ability improvements. These results suggest that, despite EHDI, most children with profound hearing loss in TSLT do not achieve age-appropriate language and literacy levels.

When describing the communication functioning prior to the commencement of EI to the communication functioning upon discharge from therapy in the LSL-SA group, the data indicate that at least two-thirds of participants achieved age-appropriate communication skills. These findings are consistent with findings by Yanbay et al. [58], who observed variability in outcomes in areas of speech and language, with some children achieving below-average scores and others achieving above-average scores. Yanbay et al. [58] also noted that family involvement in the intervention process was a key factor in significantly improving language outcomes. While the LSL-SA intervention approach emphasizes a family-based approach, which may contribute to improved outcomes, it is important to recognize that several factors, such as the intensity and consistency of the intervention, the level of family support, and individual child characteristics, also play crucial roles in influencing results. Therefore, while the data suggests that children with hearing loss enrolled in an LSL intervention approach have higher odds of performing at age-appropriate speech and language levels, the extent to which it produces better outcomes compared to other interventions must be considered within a broader context of these unaccounted variables. This highlights the need for further investigation into how these multiple factors interact and contribute to communication outcomes in children with hearing loss.

Upon comparing the communication outcomes between the two groups (i.e., TSLT and LSL-SA), the findings reveal that the LSL-SA group are more likely to achieve age-appropriate communication skills. This confirms findings from the exploratory study [51] and aligns with Percy-Smith et al. [31], who found that children with hearing loss in an LSL approach outperformed those in TSLT on speech intelligibility, expressive vocabulary, and receptive language. Percy-Smith et al. [31] adjusted the effect of the intervention approach (i.e., LSL versus TSLT) with other covariates and found that LSL participants had higher odds of reaching age-appropriate speech and language levels. The current study reinforces the effectiveness of the LSL-SA approach in achieving optimal communication outcomes. Although the LSL-SA approach demonstrates favourable outcomes, this study recognizes that the focus on spoken language interventions may inadvertently perpetuate systemic barriers to inclusive practices. The lack of resources for SASL and bilingual approaches reflects broader inequities that constrain family choices and limit the alignment of interventions with cultural and linguistic rights. The specifics of both intervention approaches, including their theoretical underpinnings, implementation processes, and individualized strategies, are critical to understanding their impact on communication outcomes. Future research should provide more granular descriptions of intervention protocols to enhance clarity and reproducibility. These results may enable policymakers, especially in LMICs, to allocate resources for and plan strategically to promote and enhance access to this early intervention approach, alleviating the deleterious consequences of hearing loss. It is, therefore, likely that the costs of hearing loss (i.e., health sector, hearing devices, educational support, loss of productivity, societal costs) on the global economy, currently at US$ 980 billion annually, may be reduced [59]. The effectiveness of the LSL-SA approach, as shown by its better outcomes when compared to TSLT in terms of achieving age-appropriate communication skills, suggests that policymakers should consider promoting and enhancing access to LSL-SA to improve communication outcomes for children with hearing loss in South Africa. It is important to acknowledge and underscore that the prioritization of spoken language interventions raises critical questions about inclusivity and equity in the context of Deaf education. Incorporating sign language and fostering bilingualism might better address these concerns, supporting a broader range of outcomes that align with the preferences of Deaf communities. These findings align with international research demonstrating that auditory-based early interventions, when accessible and appropriately implemented, can significantly improve communication outcomes. Countries with established LSL programs, such as Australia and the United States, have reported similar benefits, suggesting that structured, parent-centered interventions could be beneficial beyond the South African context. However, variations in healthcare infrastructure, cultural attitudes toward Deaf identity, and language policies must be considered when adapting such approaches to different regions.

When examining the selected study variables (i.e., age at diagnosis of hearing loss, age at amplification, duration of amplification, hearing amplification device, age at the start of EI, duration of EI, degree of the aided audiogram, home language vs. language used in therapy) within the intervention approaches, despite findings of this study showing the children enrolled in the LSL-SA cohort is characterized by a lower median age of identification of hearing loss (1.0 years old), lower median age of diagnosis of hearing loss (1.5 years old) and a lower median earliest age of amplification (1.8 years old), it highlights the incongruence between transferring the theory of the adjusted 1-4-8 South African guidelines into practice. These findings exemplify what [3,24] claim regarding the incongruence of disability in relation to the number and distribution of speech-language and hearing professionals in the South African context. These findings advocate for the training of additional therapists to address the capacity-demand ratio so that more children with hearing loss will have access to evidence-based EI services.

The age of diagnosis of hearing loss determines the age of amplification and, subsequently, the age of enrolment in a communication intervention program [3,60]. Findings of this study reveal that, despite the average age of earliest hearing aid amplification (1.8 years old) and cochlear implantation (2.7 years old) being earlier for the LSL-SA group, the age of enrolment in a communication intervention program (2.5 years old) was the same for both cohorts. This finding highlights that international and national EHDI guidelines may not be transferable to clinical practice in South Africa due to contextual constraints. Studies by [3] and Meyer et al. [61] highlight delays in amplification in South Africa. The current study’s findings advocate for the timely provision of early amplification and/or cochlear implantation and confirm that auditory input is key to communication outcomes in children with hearing loss [13,20]. These findings further motivate an increase in budget allocation to meet the demands for hearing amplification devices and may contribute toward initiatives for affordable and accessible service delivery in LMICs. As far as timing of intervention and communication outcomes, current findings highlight that early identification, diagnosis, and amplification are crucial for achieving age-appropriate communication skills. The study emphasizes the need for timely intervention to mitigate the negative impact of delayed communication skills on speech intelligibility, language development, educational outcomes, and cognitive development. Furthermore, the incongruence between guidelines and practice demonstrated by current findings shows that despite guidelines like the 1-4-8 EHDI in South Africa, there are challenges in translating these guidelines into practice, leading to delays in access to intervention services. Addressing these incongruences requires additional training for therapists and improving the capacity of healthcare systems to deliver timely interventions.

The degree of improved hearing provided by amplification devices is associated with better speech and language development in children with hearing loss [16]. However, the issue of successful amplification is multifaceted and must be carefully considered. While the current study’s findings show that the duration of hearing amplification is associated with decreased odds of delay in speech and cognitive linguistics, it is important to recognize that the mere provision of amplification does not guarantee positive outcomes. Successful amplification depends on several factors, including the appropriateness of the device, proper fitting, consistent use, and ongoing maintenance. This concurs with the findings by Tomblin et al. [16]. Additionally, access to high-quality hearing devices and the ability to maintain their use is a significant challenge in resource-constrained settings. These findings support the early provision of well-fitted hearing aids and/or cochlear implants, but they also highlight the need for a comprehensive approach to amplification. Ensuring the availability of affordable, well-fitted hearing devices, as well as providing families with the necessary support for consistent device use, is crucial for improving speech and language outcomes. Adequate budget allocation and initiatives for increasing affordability and accessibility of hearing devices are crucial for achieving optimal outcomes in this context.

Findings of the current study indicate that children in the LSL-SA approach attend therapy for a significantly shorter duration than those in TSLT. These findings concur with the exploratory study [51] and with reports indicating substantial health gains within the Disability-Adjusted Life Years (DALYs) [62]. These findings directly influence the financial, human and infrastructure resource constraints and raise implications for managing the burden of disease in South Africa.

The findings of this study show that human resource constraints in providing communication intervention services foment a linguistic mismatch between speech-language therapists and/or audiologists and the population they serve within the South African context. This concurs with the findings of numerous studies [3,24,63]. The findings of this study thus raise implications of implementing an adapted first-world intervention approach that requires contextually relevant and linguistically responsive evidence. Early communication interventionists remain accountable for acquiring reliable and valid evidence in communication approaches and need to consider resources from a cultural and linguistic perspective, as it is key to effective clinical service provision in South Africa (Health Professions Council of South Africa [64]. This study’s retrospective design limits its ability to establish causality or definitively attribute outcomes to specific interventions. A well-designed prospective study incorporating randomized controlled methods would be essential to confirm these findings and draw more robust conclusions about the comparative efficacy of LSL-SA and TSLT approaches.

Results examining whether associations exist between the selected study variables (i.e., age at diagnosis of hearing loss, age at amplification, duration of amplification, hearing amplification device, age at the start of EI, duration of EI, degree of the aided audiogram, home language vs. language used in therapy) and the communication outcomes (controlling for the communication intervention approach and type of hearing amplification device), reveal that children with hearing loss who have access to EI (which includes the diagnosis of hearing loss, fitting of hearing amplification devices, and adopting a linguistically appropriate communication approach, i.e., communication methods, resources or interventions that are tailored to the language needs and cultural background of the child) at the earliest opportunity increase their chances of achieving age-appropriate communication outcomes. This is despite the participant sample not adhering to the South African 1-4-8 EHDI guidelines. These results concur with the literature that children with earlier diagnosed hearing loss and amplification that quickly follow demonstrate more advanced language outcomes than those diagnosed and fitted with hearing amplification devices later [13,16,65]. Results further indicate that communication outcomes are hindered when the communication intervention approach does not incorporate linguistically appropriate care. This finding concurs with outcomes described in the exploratory study [51] and is particularly relevant in the South African context.

When examining the type of school recommended after EI based on communication outcomes as a predictor of success, results show that children with hearing loss enrolled in an LSL communication approach outperformed children with hearing loss enrolled in a TSLT communication approach. These findings again confirm those of [51] and Goldblatt and Pinto [66], who state that significant associations exist between language function and academic progress as an outcome of EI. Spoken language is often seen as a gateway to inclusive education and social competence [67,68]. However, it is important to note that this study’s focus on only two intervention approaches—TSLT and LSL-SA-excludes other options, such as SASL or bilingual approaches, which may better align with Deaf culture and identity and offer a more inclusive range of communication options for families. The findings, therefore, are limited in their generalizability, particularly for families who prefer or have access to alternative communication strategies. While the LSL-SA approach may support academic progress in some contexts, future research should consider the broader perspectives of the Deaf community and explore how different approaches, including sign language, can contribute to more culturally responsive, inclusive educational outcomes.

## 5. Conclusions

This study highlights the critical role of EHDI in improving communication outcomes for children with hearing loss. The findings highlight the importance of timely amplification, structured intervention approaches, and family-centered therapies in promoting age-appropriate language development. Moreover, the study emphasizes the need for context-specific solutions in LMICS, where resource constraints pose challenges to implementing optimal EHDI programs. These insights can inform policymakers and clinicians working toward improved access and inclusivity in early intervention services globally. Results from this study emphasize the importance of adapting EI communication approaches to local contexts, ensuring cultural sensitivity, linguistic responsiveness and continuity of care. To promote truly inclusive education and societal participation, there is an urgent need to integrate spoken language approaches with robust support for sign language and bilingual communication. This requires addressing systemic inequities and fostering interventions that uphold the linguistic and cultural rights of all children with hearing loss. The LSL-SA approach emphasizes family involvement, with parents playing an active role in reinforcing language learning in everyday contexts. This family-centered approach is shown to increase the odds of achieving age-appropriate communication outcomes for infants and young children with hearing loss in South Africa. However, despite its effectiveness, the LSL-SA approach faces significant barriers in South Africa. There are only 51 LSL-SA therapists and 7 LSLS Cert.AVT^®^ therapists nationwide, which limits the availability and accessibility of this intervention. The capacity–demand ratio is further compounded by the disparities between the public-private healthcare sectors, with 82.6% of the population relying on an overburdened public healthcare system, while only 17.4% have access to private healthcare [69]. Thus, the imperative of developing updated policies and guidelines for children with hearing loss in South Africa is emphasized. These policies, as well as practices, should align with inclusive frameworks that integrate diverse communication options.

Although this study was conducted in South Africa, its findings offer valuable insights for other LMICs and underserved populations globally. The evidence supporting earlier amplification, structured early intervention, and linguistic considerations can guide policymakers in designing sustainable, contextually appropriate hearing healthcare frameworks. Future research should explore how these findings translate into diverse settings, ensuring equitable access to effective communication interventions worldwide.

## 6. Limitations of the Study

When interpreting the findings of this study, several limitations must be considered. Given that this study adopted a retrospective design, randomization was not feasible. The children were grouped based on the interventions they had already received. Consequently, the assignment to one of the intervention groups was not randomized but rather reflective of the services offered by each school at the time the child’s intervention took place. This lack of randomization introduces the potential for selection bias, as factors such as the availability of resources and institutional preferences may have influenced the assignment to one of the intervention groups. To mitigate this bias, the researchers cross-validated the intervention groups with retrospective records, ensuring that children had received the intervention assigned by their school. Furthermore, the two intervention groups were matched for gender and type of amplification device, which helped reduce potential bias. However, some limitations may remain due to the non-random assignment.

Despite these limitations, the findings provide valuable context-specific evidence on communication outcomes for children with hearing loss.

## Figures and Tables

**Table 1:** Demographic data of children included in the study (N = 126).

Characteristic	Category	Overall		TSLT		LSL-SA		*p*-Value for Between-Group Comparison
		n	%	N	%	N	%	
N		126		64		62		
Age	Median (IQR); range	8.3 (6.0–10.1); 1.7–16.7		8.7 (6.5–10.2); 1.7–16.7		6.8 (5.6–10.0); 1.7–11.7		0.092
Gender	Male	54	43	29	45	25	40	0.59
	Female	72	57	35	55	37	60	
Race Group	Colored	53	42	29	45	23	37	0.61
	White	41	33	19	30	22	35	
	Black	23	18	13	20	10	16	
	Indian	7	6	2	3	5	8	
	Other	3	2	1	2	2	3	
Home Language	English	58	46	27	42	31	50	0.84
	Afrikaans	39	31	21	33	18	29	
	Xhosa	17	13	4	6	10	16	
	English and Afrikaans	5	4	10	16	7	11	
	Zulu	4	3	2	3	3	5	
	Southern Sotho	1	1	0	0	1	2	
	Other	2	2	1	2	1	2	
Type of Amplification	Hearing Aid	37	29	23	36	14	23	0.12
	Cochlear Implant	89	71	41	64	48	77	
Number of Cochlear Implants	0	36	29	22	34	14	23	0.26
	1	39	31	20	31	19	31	
	2	51	40	22	34	29	47	

**Table 2:** Comparison of Communication Functioning prior to the commencement of EI and upon discharge from EI.

Communication Domains	TSLT						LSL-SA					
	Communication Functioning at the Onset of Therapy			Communication Functioning at Discharge of Therapy			Communication Functioning at the Onset of Therapy			**Communication Functioning at Discharge of Therapy**		
	% Delayed	% Age Appropriate	% Unknown	% Delayed n	% Age Appropriate	% Unknown	% Delayed	% Age Appropriate	% Unknown	**% Delayed**	**% Age Appropriate**	**% Unknown**
Speech intelligibility	97% (n = 62)	2% (n = 1)	1% (n = 1)	55% (n = 35)	45% (n = 29)	1% (n = 1)	97% (n = 60)	3% (n = 2)		32% (n = 20)	66% (n = 41)	
Vocabulary	98% (n = 63)	2% (n = 1)		61% (n = 39)	39% (n = 25)		97% (n = 60)	3% (n = 2)		39% (n = 24)	61% (n = 38)	
Receptive Language	98% (n = 63)	2% (n = 1)		61% (n = 39)	39% (n = 25)		97% (n = 60)	3% (n = 2)		37% (n = 23)	61% (n = 38)	
Expressive Language	98% (n = 63)	2% (n = 1)		61% (n = 39)	39% (n = 25)		97% (n = 60)	3% (n = 2)		44% (n = 27)	56% (n = 35)	
Audition	98% (n = 63)	2% (n = 1)		56% (n = 36)	44% (n = 28)		98% (n = 63)	2% (n = 1)		32% (n = 20)	68% (n = 42)	
Cognitive Linguistics	98% (n = 63)	2% (n = 1)		52% (n = 33)	48% (n = 31)		90% (n = 56)	8% n = 5)	1% (n = 1)	27% (n = 17)	73% (n = 45)	

**Table 3:** Comparison of communication outcomes between the TSLT and LSL-SA groups (N = 126).

N		126		64		62			
		Overall		TSLT		LSL-SA		Unadjusted	Adjusted
Communication Outcomes Trajectory (Onset to Discharge):		N	%	n	%	n	%	Odds Ratio (OR) for LSL vs. TSLT (95% CI)	
Speech	Delayed—Delayed	54	43	34	53	20	32	0.42 (0.20–0.86)	0.46 (0.21–1.02)
	Delayed—Age-Appropriate	67	53	28	44	39	63	reference	
	Age Appropriate—Age Appropriate	3	2	1	2	2	3		
	Unknown	2	2						
Vocabulary	Delayed—Delayed	62	49	38	59	24	39	0.41 (0.20–0.83)	0.48 (0.21–1.07)
	Age Appropriate—Delayed	1	1	1	2				
	Delayed—Age-Appropriate	61	48	25	39	36	58	reference	
	Age Appropriate—Age Appropriate	2	2			2	3		
Receptive Language	Delayed—Delayed	61	48	38	59	23	37	0.38 (0.18–0.78)	0.43 (0.19–0.99)
	Age Appropriate—Delayed	1	1	1	2				
	Delayed—Age-Appropriate	62	49	25	39	37	60	reference	
	Age Appropriate—Age Appropriate	2	2			2	3		
Expressive Language	Delayed—Delayed	65	52	38	59	27	44	0.50 (0.24–1.01)	0.61 (0.28–1.33)
	Age Appropriate—Delayed	1	1	1	2				
	Delayed—Age-Appropriate	58	46	25	39	33	53	reference	
	Age Appropriate—Age Appropriate	2	2			2	3		
Audition	Delayed—Delayed	55	44	35	55	20	32	0.37 (0.18–0.77)	0.42 (0.19–0.92)
	Age Appropriate—Delayed	1	1	1	2				
	Delayed—Age-Appropriate	69	55	28	44	41	66	reference	
	Age Appropriate—Age Appropriate	1	1			1	2		
Cognitive Linguistics	Delayed—Delayed	49	39	33	52	16	26	0.33 (0.16–0.71)	0.39 (0.17–0.88)
	Delayed—Age-Appropriate	70	56	30	47	40	65	reference	
	Age Appropriate—Age Appropriate	6	5	1	2	5	8		
	Unknown	1	1						

**Table 4:** Categorical variables of participants within each intervention approach (N = 126).

Characteristic	Category	Overall		TSLT		LSL-SA		*p*-Value for Between-Group Comparison
		N	%	n	%	n	%	
N		126		64		62		
Identification and Diagnosis of Hearing Loss								
Age of identification of hearing loss (years)	median (IQR); range	1.5 (0.5–2.5); 0.0–8.1		1.7 (0.7–2.5); 0.0–7.7		1.0 (0.3–2.3); 0.0–8.1		0.047
Duration from identification to diagnosis (months)	median (IQR); range	0.0 (0.0–7.0); 0.0–58.0		0.0 (0.0–6.5); 0.0–37.0		0.5 (0.0–7.0); 0.0–58.0		0.19
Age at diagnosis of hearing loss (years)	median (IQR); range	1.8 (0.8–3.0); 0.0–8.1		2.1 (1.2–3.0); 0.1–7.7		1.5 (0.5–3.0); 0.0–8.1		0.095
Hearing Amplification								
Duration from diagnosis to amplification (months)	median (IQR); range	1.0 (0.0–3.0); 0.0–61.0		1.0 (0.0–4.0); 0.0–61.0		0.0 (0.0–2.0); 0.0–13.9		0.075
Earliest age of amplification (years)	median (IQR); range	2.1 (1.0–3.1); 0.1–8.2		2.3 (1.7–3.3); 0.1–8.2		1.8 (0.6–3.0); 0.2–8.2		0.037
Age of amplification with CI (years)	(n) median (IQR); range	(n = 90) 3.1 (1.9–4.9); 0.2–11.8		(n = 42) 3.5 (2.3–5.0); 0.2–11.8		(n = 48) 2.7 (1.8–4.9); 0.5–8.7		0.16
Age at the start of Communication Intervention (years)	median (IQR); range	2.5 (1.5–3.6); 0.2–8.3		2.5 (1.6–3.5); 0.3–8.3		2.5 (1.4–3.9); 0.2–8.2		0.86
Type of hearing amplification device	Hearing Aid	37	29	23	36	14	23	0.12
	Cochlear Implant	89	71	41	64	48	77	
Communication Intervention								
Duration from diagnosis to Communication Intervention (months)	median (IQR); range	2.7 (1.0–7.0); 0.0–67.2		2.7 (0.7–6.3); 0.0–53.6		2.9 (1.2–11.9); 0.0–67.2		0.40
Duration of Communication Intervention (years)	median (IQR); range	4.6 (3.0–6.8); 0.5–13.6		5.5 (3.4–7.3); 0.5–13.6		4.2 (2.9–6.3); 1.0–10.9		0.071
Hearing Age until discharge from Communication Intervention	median (IQR); range	4.1 (2.6–6.0); 0.1–24.0		4.4 (2.4–6.5); 0.7–24.0		4.0 (2.7–5.4); 0.1–10.7		0.61
Language of Communication Intervention								
The language used in sessions	English	89	71	43	67	46	74	0.44
	Afrikaans	37	29	21	33	16	26	
Language used during communication intervention sessions	Same	100	79	50	78	50	81	0.83
	Different	26	21	14	22	12	19	

**Table 5:** Aided Audiogram Results.

Characteristic		Overall		TSLT		LSL-SA		*p*-Value for Between-Group Comparisons
		n	%	N	%	n	%	
Aided Audiogram Results Available	No	60	48	20	31	40	65	0.0003
	Yes	66	52	44	69	22	35	
% fail (25dB cutoff): Ear R	250 Hz (n = 54)		28		29		26	>0.99
	500 Hz (n = 58)		40		40		40	>0.99
	1000 Hz (n = 58)		43		42		45	>0.99
	2000 Hz (n = 56)		55		58		50	0.77
	4000 Hz (n = 49)		63		71		43	0.10
	8000 Hz (n = 7)							
	Low frequencies (250–1000 Hz) (n = 58)		48		45		55	0.58
	High frequencies (2000–4000 Hz) (n = 56)		68		74		56	0.23
% fail (25dB cutoff): Ear L	250 Hz (n = 54)		30		31		28	>0.99
	500 Hz (n = 56)		43		46		37	0.58
	1000 Hz (n = 56)		41		38		47	0.57
	2000 Hz (n = 55)		45		49		39	0.57
	4000 Hz (n = 49)		49		56		33	0.22
	8000 Hz (n = 5)							
	Low frequencies (250–1000 Hz) (n = 56)		50		51		47	>0.99
	High frequencies (2000–4000 Hz) (n = 55)		60		65		50	0.38

**Table 6:** Association between the categorical variables and communication outcomes.

Odds Ratios are for Delay vs. Age Appropriate		Speech			Vocabulary			Receptive Language			Expressive Language			Audition			**Cognitive Linguistics**		
		Delayed	Age Appropriate	OR (95% CI)	Delayed	Age Appropriate	OR (95% CI)	Delayed	Age Appropriate	OR (95% CI)	Delayed	Age Appropriate	OR (95% CI)	Delayed	Age Appropriate	OR (95% CI)	**Delayed**	**Age Appropriate**	**OR (95% CI)**
Duration from ID to diagnosis (m)	median (IQR)	0.0 (0.0–11.0)	0.0 (0.0–3.0)	1.04 (1.01–1.08)	0.0 (0.0–9.0)	0.0 (0.0–3.0)	1.03 (0.99–1.07)	0.0 (0.0–9.0)	0.0 (0.0–3.0)	1.04 (0.99–1.08)	0.0 (0.0–8.0)	0.0 (0.0–3.0)	1.03 (0.99–1.07)	0.0 (0.0–8.0)	0.0 (0.0–6.0)	1.01 (0.98–1.05)	0.0 (0.0–8.0)	0.0 (0.0–6.0)	1.01 (0.98–1.05)
Duration from ID to EI (m)	median (IQR)	8.2 (2.6–22.7)	6.0 (2.6–19.2)	1.02 (0.99–1.04)	7.4 (2.7–18.3)	6.9 (2.5–26.3)	1.00 (0.98–1.03)	7.2 (2.7–16.9)	7.2 (2.5–26.9)	1.00 (0.97–1.02)	7.8 (2.8–18.3)	6.5 (2.4–23.8)	1.00 (0.98–1.03)	6.9 (2.6–14.4)	7.5 (2.6–27.6)	0.99 (0.96–1.02)	7.8 (2.6–18.3)	6.6 (2.7–23.8)	1.00 (0.97–1.03)
Duration from identification to amplification (m)	median (IQR); range	6.0 (1.0–15.0)	2.0 (0.0–8.5)	1.03 (0.99–1.07)	6.0 (1.0–18.0)	2.0 (0.0–8.0)	1.04 (0.99–1.07)	6.0 (1.0–18.0)	2.0 (0.0–7.6)	1.04 (0.99–1.07)	6.0 (1.0–15.0)	2.0 (0.0–8.3)	1.03 (0.98–1.07)	6.0 (1.1–12.5)	2.0 (0.0–9.0)	1.01 (0.98–1.05)	6.0 (1.1–12.0)	3.0 (0.0–9.0)	1.01 (0.98–1.05)
Duration of Amplification (y)	median (IQR); range	3.4 (1.8–5.4)	4.9 (3.5–6.4)	0.73 (0.59–0.89)	3.9 (2.3–5.7)	4.7 (3.4–6.3)	0.86 (0.72–1.01)	3.9 (2.3–5.7)	4.7 (3.3–6.2)	0.88 (0.74–1.04)	3.9 (2.3–5.7)	4.8 (3.4–6.3)	0.86 (0.72–1.02)	3.8 (2.3–5.5)	4.7 (3.2–6.3)	0.85 (0.71–1.02)	3.4 (1.8–5.4)	4.8 (3.4–6.3)	0.76 (0.62–0.93)
Audiogram (% fail)	Ear A—low frequencies (n = 58)	63	38	3.03 (0.96–9.53)	63	38	3.10 (0.96–10.0)	61	40	2.79 (0.84–9.22)	63	38	3.10 (0.96–10.0)	60	42	2.50 (0.71–8.85)	72	38	6.14 (1.48–25.4)
	Ear A—high frequencies (n = 56)	83	58	2.67 (0.71–10.1)	87	55	4.11 (0.97–17.5)	86	56	3.49 (0.81–15.1)	87	55	4.11 (0.97–17.5)	90	56	5.08 (0.95–27.1)	94	55	10.3 (1.19–89)
	Ear B—low frequencies (n = 56)	67	38	3.31 (1.06–10.4)	67	38	3.49 (1.05–11.6)	65	39	3.08 (0.90–10.5)	67	38	3.49 (1.05–11.6)	63	43	2.52 (0.68–9.38)	71	41	3.83 (1.01–14.6)
	Ear B—high frequencies (n = 56)	75	48	2.69 (0.78–9.32)	78	47	2.57 (0.69–9.52)	77	48	2.07 (0.54–7.92)	78	47	2.57 (0.69–9.52)	79	50	2.09 (0.50–8.71)	88	47	6.22 (1.16–33)
Number of cochlear implants (column %)	0	34	23	reference	41	16	reference	42	16	reference	41	15	reference	46	14	reference	46	17	reference
	1	31	31	0.73 (0.28–1.92)	37	25	0.58 (0.22–1.58)	35	27	0.53 (0.20–1.42)	35	27	0.50 (0.19–1.36)	36	27	0.43 (0.16–1.14)	42	24	0.71 (0.27–1.45)
	2	34	47	0.14 (0.05–0.38)	22	59	0.16 (0.06–0.41)	23	58	0.16 (0.06–0.42)	24	58	0.16 (0.06–0.42)	18	59	0.10 (0.04–0.28)	12	59	0.37 (0.16–0.86)
Age at start of EI (y)	median (IQR); range	3.0 (2.1–4.3)	2.3 (1.0–3.3)	1.49 (1.15–1.92)	2.9 (2.1–4.2)	2.1 (0.9–3.1)	1.52 (1.16–1.99)	2.9 (2.1–4.2)	2.2 (0.9–3.1)	1.52 (1.16–1.99)	2.8 (2.1–4.1)	2.1 (0.9–3.2)	1.47 (1.13–1.90)	2.9 (1.9–4.3)	2.3 (1.0–3.3)	1.38 (1.07–1.78)	3.0 (2.1–4.5)	2.3 (1.0–3.2)	1.50 (1.16–1.96)
Earliest age of amplification (y)	median (IQR); range	2.8 (1.8–4.0)	1.8 (0.6–2.6)	1.65 (1.24–2.21)	2.8 (2.0–4.0)	1.4 (0.4–2.4)	2.07 (1.47–2.92)	2.8 (2.0–4.0)	1.4 (0.5–2.4)	2.18 (1.53–3.11)	2.6 (1.8–4.0)	1.5 (0.4–2.5)	1.88 (1.35–2.60)	2.8 (2.0–4.0)	1.6 (0.6–2.6)	1.75 (1.27–2.39)	2.9 (2.0–4.2)	1.8 (0.6–2.6)	1.74 (1.28–2.37)
Earliest age of amplification with CI (y) (n = 90)	median (IQR); range	4.0 (3.0–5.7)	2.5 (1.6–4.2)	1.34 (1.07–1.68)	3.6 (2.5–5.2)	2.7 (1.6–4.3)	1.19 (0.97–1.46)	3.6 (2.5–5.1)	2.8 (1.6–4.5)	1.17 (0.95–1.43)	3.6 (2.3–5.2)	2.8 (1.6–4.5)	1.15 (0.95–1.40)	3.6 (2.5–5.3)	2.8 (1.7–4.7)	1.15 (0.94–1.41)	4.3 (3.0–5.3)	2.7 (1.7–4.3)	1.23 (0.99–1.52)
Earliest age of amplification vs. Age at start of EI(cutoffs 2.1y and 2.5y respectively	Early / Early	36	59	reference	41	57	reference	42	56	reference	42	57	reference	41	56	reference	36	58	reference
	Early / Late	5	16	0.83 (0.20–3.47)	3	19	0.33 (0.07–1.70)	2	20	0.16 (0.02–1.3)	5	18	0.46 (0.11–1.90)	2	19	0.20 (0.02–1.75)	4	16	0.68 (0.13–3.59)
	Late / Late	58	26	3.38 (1.49–7.66)	56	24	2.97 (1.30–6.75)	56	23	2.97 (1.30–6.78)	53	25	2.55 (1.13–5.78)	57	26	2.75 (1.20–6.62)	60	26	3.42 (1.48–7.94)
Duration of EI (y)	median (IQR); range	4.1 (2.4–6.8)	5.4 (3.8–7.3)	0.81 (0.68–0.95)	4.1 (2.9–6.8)	5.4 (3.6–7.4)	0.80 (0.68–0.94)	4.2 (2.9–6.8)	5.4 (3.6–7.3)	0.81 (0.68–0.95)	4.1 (2.9–6.6)	5.5 (3.8–7.4)	0.79 (0.67–0.93)	4.1 (2.8–6.7)	5.4 (3.7–7.3)	0.77 (0.65–0.91)	3.7 (2.8–6.1)	5.5 (3.8–7.3)	0.72 (0.60–0.87)
SLT Language match to home language	Same	67	89	reference	67	92	reference	66	92	reference	67	93	reference	61	94	reference	62	91	reference
	Different	33	11	3.55 (1.34–9.41)	33	8	5.69 (1.88–17.2)	34	8	6.06 (1.98–18.5)	33	7	6.74 (2.08–21.9)	39	6	12.3 (3.57–42.6)	38	9	6.28 (2.20–17.9)

## Data Availability

All data relevant to the study are included in the article.

## Abbreviations

The following abbreviations are used in this manuscript:

LSL-SA	Listening and Spoken Language-South Africa
TSLT	Traditional Speech-Language Therapy
EHDI	Early Hearing Detection and Intervention
NHS	Newborn Hearing Screening
EI	Early Intervention
AVT	Auditory Verbal Therapy
LMIC	Low-middle-income country
ECD	Early Childhood Development
SASL	South African Sign Language
NDP	National Development Plan
HPCSA	Health Professions Council of South Africa
CI	Cochlear Implant
DALYs	Disability-Adjusted Life Years
